# Flow cytometry of non-hematopoietic cells in canine effusions

**DOI:** 10.3389/fvets.2024.1414271

**Published:** 2024-09-24

**Authors:** Federica Sini, Maverick Melega, Francesca Tiziana Cannizzo, Barbara Miniscalco, Paola Valenti, Fulvio Riondato

**Affiliations:** ^1^Department of Veterinary Sciences, School of Agriculture and Veterinary Medicine, University of Turin, Grugliasco, TO, Italy; ^2^Clinica Veterinaria Malpensa AniCura, Samarate, VA, Italy

**Keywords:** effusion, cell block, flow cytometry, vimentin, cytokeratin, desmin, carcinoma, mesothelioma

## Abstract

The identification of non-hematopoietic cells in effusions is a diagnostic challenge in cytology. Biopsies from mesothelium or primary lesions are infrequently performed in clinical settings and immunochemistry on smears or immunohistochemistry on cell blocks are the most common ancillary test to refine the cytological diagnosis. Cavitary effusions are an ideal matrix for flow cytometry and the availability of a cytometric panel to describe non-hematopoietic cells would represent a useful tool. Here we present the results of the flow cytometric and immunohistochemical determination of cytokeratin (CK), vimentin (VIM) and desmin (DES) in 36 canine effusions. The concordance between the two methods was perfect for CK (100%), substantial for VIM (77.8%), and almost perfect for DES (97.2%). The panel was interpreted to define the epithelial (CK+VIM-DES-), mesothelial (CK+VIM+DES+), or mesenchymal (CK-VIM+DES-) origin of the cells. Unexpected profiles were considered doubtful and observed patterns were individually discussed. The concordance of the panel interpretation between two methods was 75%. The evaluation of discordant and doubtful cases suggests a lower sensitivity of flow cytometry in detecting VIM expression and revealed a high frequency of VIM+ epithelial cells, variable expression of VIM in mesothelial cells, and an important role of DES in excluding an epithelial origin when positive. Multicentric studies based on histopathological diagnoses are necessary to confirm these findings and evaluate the diagnostic utility of the panel to refine cytological diagnosis. Our results show that flow cytometry can be a timesaving alternative to IHC on cell blocks in clinical settings to detect CK, VIM and DES expression. The interpretation of the panel is similar in most cases; however, occasional discordant results, particularly for VIM, may occur.

## 1 Introduction

Effusion cytology is complex as multiple inflammatory, reactive, and possibly neoplastic cells may be present in the same sample. Main challenges include the identification of cells based on the shape (cells in fluid appear round regardless of their origin), the presence of mesothelial cells that readily exfoliate regardless the underlying cause, and the overlapping morphology of reactive mesothelial cells, neoplastic mesothelial cells and other exfoliative malignant cells (e.g., neoplastic epithelial cells). The presence of cohesive clusters can suggest an epithelial origin; however, both reactive and neoplastic mesothelial cells can exfoliate in variably cohesive aggregates. Also, poorly differentiated carcinomas can be less cohesive with individualized cells predominating (1).

Whilst cytology can be useful to classify transudate and inflammatory effusions, its sensitivity for the diagnosis of malignancy is limited, particularly in case of non-hematopoietic (NH) cells (2–4). In these cases, a final diagnosis can be achieved with histopathology and possibly immunohistochemistry (IHC) of the primary lesions. However, fluid collection and cytology are often not followed by more invasive diagnostic procedures; the collection of intrathoracic or abdominal tissue biopsies is often declined by pet owners or is clinically not recommended given the unstable condition of some of these patients. Ancillary techniques can be used to refine the cytological diagnosis by defining the immunophenotype, and therefore the origin, of NH cell in cavitary effusions. In veterinary medicine, immunochemistry on cytological preparations (5) and IHC on cell blocks have been described and successfully applied on effusions for immunochemical characterization (4, 6–9).

Currently, the minimum panel to differentiate epithelial and mesothelial cells should include cytokeratin (CK) and vimentin (VIM) (10). Co-expression of CK and VIM is considered the main feature of mesothelial cells, while expression of CK or VIM only, is expected in epithelial and mesenchymal cells, respectively (11). However, several reports revealed variable VIM expression in epithelial and mesothelial cells (4, 7). To overcome this limitation, a wider panel including expected positive and negative markers has been recommended for the diagnosis of malignant mesothelioma in human medicine (12). Desmin (DES) is used to distinguish reactive mesothelial (DES+) from neoplastic mesothelial and epithelial cells (DES-) in people (13, 14). In veterinary medicine, DES does not appear to be an exclusive marker to distinguish reactive from neoplastic mesothelial cells, but it has been proven useful in differentiating mesothelial and epithelial cells (4, 5, 15). A combination of CK, VIM and DES can represent a starting panel to differentiate cell lineage in effusions. Immunochemical techniques and cell block production have limitations, such as the need for additional training and significant amount of hand-on technician time, long turnaround time, and limited possibility of multi-marker analysis (6, 16, 17). Conversely, flow cytometry (FC) is a sensitive, fast, and affordable method to study fluid matrices. It allows the simultaneous analysis of multiple antigens on a high number of cells if compared with IHC and allows the characterization of subsets of cells in a mixed population. However, it does not allow for retrospective studies as the analysis is limited to fresh samples and cell viability. In human medicine, several studies demonstrated that FC can contribute to refine the cytological diagnosis of non-hematopoietic disorders in effusions (17–19). While FC is routinely used in veterinary medicine for immunophenotyping of hematological disorders in peripheral blood, bone marrow, lymph nodes, peripheral tissues, and body fluids (20–23), no data are available about the use of this technique to characterize NH cells in cavitary effusions.

The aim of this study is to compare FC determination of CK, VIM and DES in NH cells in canine effusions with paired IHC results on cell blocks. The final goal is to provide an additional tool to characterize NH cells in effusions.

## 2 Materials and methods

Canine pleural, pericardial and peritoneal cavitary effusions received at the Laboratory for Clinical Analyses of the Veterinary Teaching Hospital of the University of Turin (Grugliasco, IT) were considered for the study. Dogs were privately owned and underwent sampling for diagnostic purposes with signed informed consent from the owners. Thus, specific formal approval by the authors' Institution Committee for Animal Care was not required (protocol 1965–2017, Ethical Committee, University of Turin). Samples were collected in EDTA tubes and routinely processed. Samples with cytological evidence of cells of suspected NH origin and samples with abundant reactive mesothelial cells were included in the study if at least 2 ml of fluid were available after routine analysis. Samples were processed for FC and cell blocks were prepared within 24 h from collection. Cases with inadequate cell blocks for IHC and samples with <1% of CD45-negative or large CD11b-negative population in FC were excluded.

### 2.1 Flow cytometry

FC analysis was performed with a BD Accuri C6 (Becton Dickinson, San Josè, CA) and a Cytoflex (Beckman Coulter, Brea, USA) flow cytometer.

NH cells were detected as CD45-negative (13 cases) or large CD11b-negative events (23 cases). We previously assessed the co-expression of the two markers on eight cases showing that the two labeling allow the detection of the same population (Supplementary Figure 1).

The cellularity of the sample was assessed by flow cytometry after removal of erythrocytes with an ammonium chloride-base buffer (1:10 dilution, 10 min incubation). The quality of the sample was assessed by further addition of 10 uL of propidium iodide. A tube with ~60 × 10^5^ cells was incubated 20 min at 4°C in the dark with previously titrated anti-CD11b or anti-CD45 monoclonal antibody. Erythrocytes were lysed as described above and cells were washed with PBS by centrifuging at 1,200 rpm for 5 min. The cell pellet was processed for cytoplasmic staining using a commercial kit (eBioscienceTM Intracellular Fixation & Permeabilization Buffer Set, ThermoFisher). Briefly, it was then incubated for 10 min at 4°C with fixation buffer, washed once with PBS and twice with permeabilization buffer, resuspended in 240 uL of permeabilization buffer and the obtained volume split in six tubes. Four tubes were used for direct staining: negative control (added with 10 uL of PBS) to set the autofluorescence, isotype control, CK and VIM according to previous titration. Two tubes were used for indirect staining adding 10 ul of PBS and anti-DES monoclonal antibody, respectively. Samples were incubated for 30 min at 4°C and washed twice with permeabilization solution. Tubes for direct staining were resuspended in PBS and immediately acquired at the cytometer. Tubes for indirect staining were incubated for an additional 20 min at 4°C with AlexaFluor488-conjugated secondary antibody, washed with permeabilization buffer, resuspended in PBS, and acquired. Information about the used antibodies is reported in Table 1.

**Table 1:** List of primary and secondary antibodies used in flow cytometry and immunohistochemistry.

**Antibody**	**Clone**	**Source**	**Conjugation**	**Method**
CD45	YKIX716.13	BioRad	AlexaFluor647	FC
CD11b	M1/70	Abcam	PE-Cy5	FC
CK	CK AE1 AE3	NovusBiologicals	AlexaFluor488/Unconjugated	FC/IHC
VIM	V9	NovusBiologicals	AlexaFluor488/Unconjugated	FC/IHC
Isotypic control (Anti-IgG1K)	—	R&D Systems	AlexaFluor488	FC
DES	DER-II	Novocastra	Unconjugated	FC/IHC
Secondary antibody	—	Invitrogen	AlexaFluor488	FC

CK, cytokeratin; VIM, vimentin; DES, desmin; FC, flow cytometry; IHC, immunohistochemistry on cell block.

A minimum of 1,000 large CD11b-negative or CD45-negative events were acquired for each tube. A first gate was set in an FSC-H vs. FSC-A scattergram to exclude doublets and a second morphological gate (FSC-A vs. SSC-A) to exclude events smaller than small lymphocytes. NH cells were gated as large CD11b-negative or CD45-negative events and the positive gate was depicted to include <1% of the events in negative controls (Supplementary Figure 2). Immunoreaction to cytoplasmic markers (CK, VIM, DES) was defined positive when at least 20% of the population fell in the positive gate. All cases were analyzed by the same pathologist (FR), who was blind to IHC results.

### 2.2 Cell blocks and immunohistochemistry

Cell tube blocks were prepared as previously described (6). H&E-stained sections were assessed for presence of target cells with adequate morphology and cellularity. Cell blocks deemed adequate for IHC were further processed and stained for CK, VIM and DES. Briefly, four micrometer sections were cut, placed on Tomo^®^ IHC adhesive glass slides (Matsunami glass Ltd.) and dried in convection oven at 50°C for 30 min. IHC were performed in one session with an automated immunostainer (BenchMark XT processor, Ventana Medical Systems, Tucson, AZ). Sections were deparaffinized with xylene and rehydrated with decreasing concentrations of ethanol. Endogenous peroxidase activity was inhibited with a peroxide hydrogen 3% solution and heat induced antigen retrieval was performed with CC1 solution (EDTA) for 24 min at 100°C. Incubation was performed at 37°C for 30 min for all antibodies. Antibodies' clones were the same used in FC analysis (Table 1). The Ventana ultraView Universal DAB Detection kit was used for all samples. Histological section of canine intestine, liver, pancreas, spleen, and lymph node were used as controls.

IHC interpretation was performed reviewing May-Grunwald Giemsa cytological preparation and H&E-stained cell blocks to ensure a proper identification of NH-cells and assess the immunoreaction. Sections were assessed for proportion of NH positive cells providing a percentage from 0 to 100. The NH population was defined positive when more than 20% of the cells were positive. All samples were evaluated by the same pathologist (FTC).

### 2.3 Panel interpretation

Panels were interpreted for both methods based on the expected staining patterns for epithelial cells (CK+VIM-DES-), mesothelial cells (CK+VIM+DES+) and mesenchymal cells (CK-VIM+DES-), according to the most frequent presentation (4, 5, 10, 24). Patterns deviating from what expected were interpreted as “doubtful”.

### 2.4 Statistical analysis

FC results for each parameter (CK, VIM, DES) are reported in the text as median percentage and range (minimum–maximum). Agreement between FC and IHC results was calculated for the expression (positive or negative) of the individual markers and for the panel interpretation. The degree of agreement was defined according to the kappa value as previously reported (25): poor (0–0.20), fair (0.21–0.40), moderate (0.41–0.60), substantial (0.61–0.80), or almost perfect (0.81–1.00).

## 3 Results

Thirty-six samples from the pleural (N = 19), peritoneal (N = 11) and pericardial (N = 6) cavities from 36 dogs were included. Patients were 17 females (9 neutered) and 19 males (3 neutered), the mean age was 9.7 years (range 4–15 years).

Results from routine fluid analysis including cytology, total nucleated cell count, total solids and final diagnostic interpretation based on clinical and clinical-pathological data are reported in Supplementary Table 1.

### 3.1 Flow cytometry

Details of the individual cases are shown in Table 2, Supplementary Table 1. The median proportion of NH cells was 13.1% (range 1.1%−65.3%). Thirty-five out of 36 samples were CK positive. The median proportion of positive target cells was 93.2% (range 45.3%−99.5%). One sample was CK negative (0.3% of the target population). Eighteen out of 36 samples were VIM positive (median 66.3%; range 21.4%−98.9%). Eighteen out of 36 samples were VIM negative (median 7.8%; range 0.4%−18.2%). Sixteen out of 36 samples were DES positive (median 76%; range 34%−96.2%). Twenty out of 36 samples were DES negative (median 4.3%; range 0.1%−18.2%).

**Table 2:** Cytokeratin, vimentin, and desmin results reported by flow cytometry and immunohistochemistry on cell blocks and interpretation of the panel.

	**CK**		**VIM**		**DES**		**Panel interpretation**	
**CASE**	**FC**	**IHC**	**FC**	**IHC**	**FC**	**IHC**	**FC**	**IHC**
1	Pos	Pos	Neg	Pos	Pos	Pos	D	M
2	Pos	Pos	Neg	Pos	Pos	Pos	D	M
3	Pos	Pos	Pos	Pos	Pos	Pos	M	M
4	Pos	Pos	Pos	Pos	Neg	Neg	D	D
5	Pos	Pos	Neg	Pos	Neg	Neg	E	D
6	Neg	Neg	Pos	Pos	Neg	Neg	S	S
7	Pos	Pos	Neg	Pos	Pos	Pos	D	M
8	Pos	Pos	Neg	Neg	Neg	Neg	E	E
9	Pos	Pos	Pos	Pos	Neg	Neg	D	D
10	Pos	Pos	Pos	Pos	Neg	Neg	D	D
11	Pos	Pos	Pos	Pos	Pos	Pos	M	M
12	Pos	Pos	Pos	Pos	Pos	Pos	M	M
13	Pos	Pos	Pos	Pos	Pos	Pos	M	M
14	Pos	Pos	Neg	Neg	Neg	Neg	E	E
15	Pos	Pos	Pos	Pos	Pos	Pos	M	M
16	Pos	Pos	Pos	Pos	Neg	Neg	D	D
17	Pos	Pos	Pos	Pos	Pos	Pos	M	M
18	Pos	Pos	Neg	Neg	Neg	Neg	E	E
19	Pos	Pos	Neg	Pos	Neg	Neg	E	D
20	Pos	Pos	Pos	Pos	Neg	Neg	D	D
21	Pos	Pos	Pos	Pos	Neg	Neg	D	D
22	Pos	Pos	Pos	Pos	Pos	Pos	M	M
23	Pos	Pos	Neg	Pos	Neg	Neg	E	D
24	Pos	Pos	Pos	Pos	Pos	Pos	M	M
25	Pos	Pos	Neg	Neg	Neg	Neg	E	E
26	Pos	Pos	Neg	Neg	Pos	Neg	D	E
27	Pos	Pos	Neg	Neg	Neg	Neg	E	E
28	Pos	Pos	Neg	Pos	Pos	Pos	D	M
29	Pos	Pos	Pos	Pos	Pos	Pos	M	M
30	Pos	Pos	Neg	Neg	Neg	Neg	E	E
31	Pos	Pos	Pos	Pos	Pos	Pos	M	M
32	Pos	Pos	Neg	Neg	Neg	Neg	E	E
33	Pos	Pos	Neg	Pos	Neg	Neg	E	D
34	Pos	Pos	Pos	Pos	Pos	Pos	M	M
35	Pos	Pos	Neg	Neg	Neg	Neg	E	E
36	Pos	Pos	Neg	Neg	Neg	Neg	E	E

CK, cytokeratin; VIM, vimentin; DES, desmin; FC, flow cytometry; IHC, immunohistochemistry; Pos, positive; Neg, negative; E, epithelial; M, mesothelial; D, doubtful. Discordant results between FC and IHC are underlined.

According to the panel interpretation, NH cells were mesothelial in 11 cases (CK+VIM+DES+), epithelial in 13 cases (CK+VIM-DES-), mesenchymal in 1 case (CK-VIM+DES-) and doubtful in 11 cases (6 CK+VIM+DES- and 5 CK+VIM-DES+).

### 3.2 Immunohistochemistry

Details of the individual cases are shown in Table 2, Supplementary Table 1. Thirty-five out of 36 samples were CK positive. The median proportion of positive target cells was 91% (range 72%−100%). In one sample no CK positive cells were present. Twenty-six out of 36 samples were VIM positive (median 87%; range 21%−100%) while 10 samples were VIM negative (median 2.5%; range 0%−15%). Fifteen out of 36 samples were DES positive (median 78%; range 25%−92%) and 21 were DES negative (median 3%; range 0%−15%).

According to the panel interpretation, NH cells were mesothelial in 15 cases (CK+VIM+DES+), epithelial in 10 cases (CK+VIM-DES-), mesenchymal in 1 case (CK-VIM+DES-), and doubtful in 10 cases (CK+VIM+DES-).

### 3.3 Agreement between FC and IHC results

FC and IHC reported 36 CK concordant results (35 CK+ and 1 CK-) with 100% agreement.

**Figure 1:**
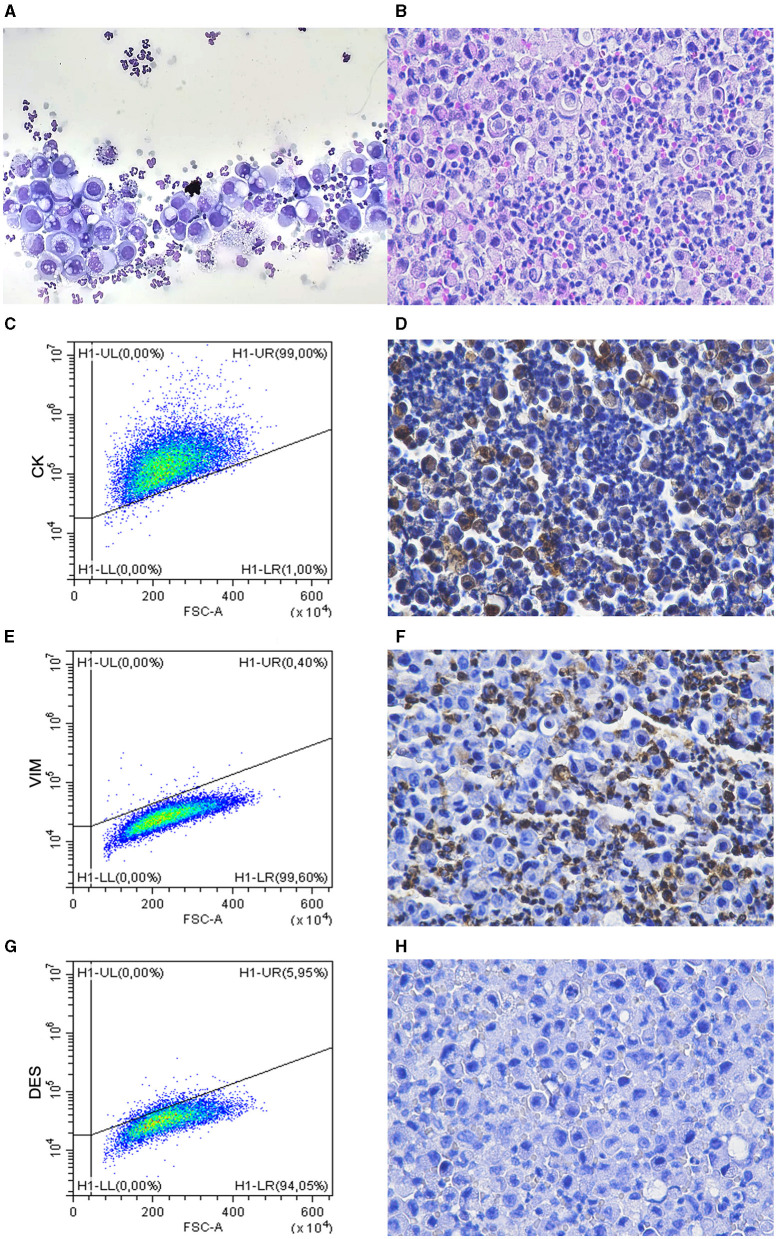
Cytology and FC results compared with H&E and IHC on cell blocks. Case 25. **(A)** Cytology. A population of large often vacuolated cells is present along with neutrophils and occasional macrophages. MGG stain. **(B)** Cell tube block. The two main population detected at cytology are recognized. H&E stain. **(C–H)** Flow cytometry **(C, E, G)** and IHC **(D, F, H)** showing NH are cells positive for CK **(C, D)** and negative for VIM **(E, F)** and DES **(G, H)**. Only CD45-negative cells are gated in flow cytometric analysis. Positive cells in **(F)** are neutrophils [negative in **(D, H)**].

The two methods reported 28 concordant (18 positive and 10 negative) and 8 discordant VIM results with a 77.8% agreement. All discordant cases were VIM- in FC and VIM+ in IHC on cell block. The percentage of positive events in FC was <10% in six cases, 17.4% and 18.2% in the remaining two. The percentage of positive cells in IHC was >70% in all but two cases (21% and 32%). FC and IHC reported 35 concordant (15 DES+ and 20 DES-) and one DES discordant results with a 97.2% agreement. The discordant case was DES+ in FC (34% of positive events) and DES- in IHC (15% of positive cells). Representative IHC pictures and FC scatterplots are shown in Figure 1.

The panel interpretation was concordant in 27/36 cases (11 mesothelial; 9 epithelial; 1 mesenchymal; 6 doubtful) with a 75% agreement. Among nine discordant cases, five were interpreted as doubtful in FC and mesothelial (4) or epithelial (1) in IHC on cell blocks. While four cases were epithelial in FC and doubtful in IHC. In 8/9 cases the discrepancy was due to VIM+ in IHC and VIM- in FC, in one case the discrepancy was due to DES- in IHC and DES+ in FC.

## 4 Discussion

Ancillary techniques such as immunocytochemistry and IHC on cell blocks are useful to refine the cytological diagnosis of effusions (10). Despite being routinely used for hematological malignancies in dogs and cats (26), the applications of FC in immunophenotyping NH cells in effusions has not been investigated in veterinary medicine. Here we describe for the first time a flow cytometric approach to immunophenotype NH cells in canine cavitary effusions.

FC requires cells to be in a suspension to be analyzed and body fluids are a “ready-to-use” matrix for this technique, making it a fast and cost-effective method to study effusions and a potential alternative to immunocytochemistry on smears and IHC on cell blocks. In human pathology, FC is being increasingly used to immunophenotype NH cells in effusions with promising results (17–19). FC allows the identification of subpopulations of cells based on morphological properties (i.e., size and complexity) and the use of combination of markers. Here, CD45 or CD11b where used to exclude hematopoietic cells from the analysis and to identify NH cells. Their phenotype was then described based on the immunoreaction to antibodies against three intermediate filaments (CK, VIM and DES). This approach allowed the analysis of samples even in the presence of low percentages of NH cells.

The agreement between FC and IHC in the interpretation of the individual markers was perfect for CK, almost perfect for DES, and substantial for VIM leading to substantial agreement in the final interpretation of the panel. The one case with discordant panel interpretation due to DES led to an epithelial classification in IHC (CK+VIM-DES-) and doubtful in FC (CK+VIM-DES+). This case was a pleural effusion suspected to be mesothelial-neoplastic in cytology; unfortunately, a definitive diagnosis was not available. All the other discordant results were due to a positive VIM reaction in IHC and negative in FC. Half of these cases were CK+VIM+DES- in IHC, interpreted as doubtful, and CK+VIM-DES- in FC, interpreted as epithelial. In this study the profile CK+VIM+DES- was considered doubtful, as possible interpretation include DES- mesothelial cells or VIM+ epithelial cells. DES- mesothelial cells have been previously reported (4, 15); however, this was considered unlikely in these cases. The epithelial origin was further supported by clinical, cytological and/or histopathological diagnosis of carcinoma (2 mammary carcinomas with multiorgan dissemination, 1 lung carcinoma, 1 gastric carcinoma). In these cases, although FC provided the expected phenotype for epithelial cells, a genuine expression of VIM was considered most likely given the strong and specific stain in IHC. VIM+ epithelial cells have been previously reported in effusions (4, 5, 7) and in some carcinomas (27). Variable VIM expression in neoplastic epithelial cells may results from type three epithelial-mesenchymal transition (EMT), where cells lose polarization and stability, gain migratory traits, and increase VIM while decreasing epithelial adhesion proteins like cadherins (28). The reason for the non-recognition of VIM in FC remains to be established and multiple factors may be contributing. NH cells aggregation may have affected permeabilization and prevented antigen-antibody binding. VIM expression may have been too low for detection by FC, where signal brightness correlates with the total amount of antigen in each cell, unlike in IHC, where staining intensity and cytoplasmatic pattern are independent parameters. All these cases were DES-, reinforcing the hypothesis that DES is negative in epithelial cells and suggesting that DES positivity could help to exclude an epithelial origin. In the remaining half of the cases NH cells were CK+VIM+DES+ in IHC, interpreted as mesothelial, and CK+VIM-DES+ in FC, interpreted as doubtful. The profile CK+VIM-DES+ was considered doubtful, as possible interpretation include VIM- mesothelial cells or DES+ epithelial cells. The presence of DES+ epithelial cells has been reported in a small proportion of cells (1–25%) in the effusion of a dog with carcinoma (4). This possibility was considered less likely here as cytology, clinical, imaging, follow-up data were strongly supportive of reactive mesothelial origin of these cells in 3 out of 4 cases; a final clinical diagnosis of ascites due to congestive heart failure, idiopathic pericarditis (alive after 2 years, no relapse) and hemorrhagic pericardial effusion due ruptured right atrial mass consistent with hemangiosarcoma. Unfortunately, insufficient evidence for a definitive diagnosis was available in one case; this was a suspected mesothelioma based on cytology alone; the patients had recurrent pleural effusion with no evidence of a primary lesion on imaging and was euthanized 3 months after presentation, necropsy was declined. These findings support the current evidence that mesothelial cells variably express VIM, ranging from negative to strongly positive (4, 7, 15) and consolidate the hypothesis that lack of VIM does not exclude a mesothelial origin.

Overall, these findings confirm that the co-expression of CK and VIM alone is not reliable in distinguishing between epithelial and mesothelial cells in effusions as previously reported (4) and that a wider panel of markers is necessary. For instance, WT-1 (4, 7) and DES appear to be good candidates for this purpose. Looking at IHC as the reference method, the discrepancies between the two techniques suggest a lower reliability of FC in detecting VIM expression but the addition of DES is useful to rule out an epithelial origin. Further studies investigating different clones to detect VIM expression in FC may be indicated.

Whilst the presence itself of epithelial or mesenchymal cells in the effusion is a strong indicator of neoplasia, further characterization is needed to distinguish between reactive and neoplastic mesothelial cells. Based on the data available in this series, VIM was variably expressed in both suspected reactive and neoplastic mesothelial cells and a possible role of VIM expression in the differentiation between these two is unlikely. In people, DES is mainly used as to distinguish reactive (DES+) from neoplastic (DES-) mesothelial cell (13, 14), while in dogs it appears to be limited to distinguish between mesothelial and epithelial cells (4, 5, 15). In this cohort, six cases showed a doubtful DES- profile both by FC and IHC. Final clinical-pathological interpretation was indicative of neoplastic effusion in five of these cases; however, the limited number of cases and lack of a definitive diagnosis hamper any solid association between the lack of DES and neoplasia. A previously reported, it is likely that DES has lower sensitivity and specificity in dogs than in people to distinguish between reactive and neoplastic mesothelial cells (4, 5, 15); this also suggests that its utility in dogs is most likely limited to distinguish mesothelial and epithelial cells. To accurately differentiate between reactive and neoplastic mesothelial cells, additional markers for cell lineages are necessary. In humans, guidelines recommended using an IHC panel including at least two mesothelial and two epithelial markers, along with epithelial membrane antigen (EMA), glucose transporter 1 (GLUT1) and insulin-like growth factor II mRNA-binding protein 3 (IMP3) to distinguish between mesothelioma and reactive hyperplasia (12). Few of these markers have been tested in dogs for similar diagnostic purposes, such as EMA (29, 30), Calretinin (29, 31–36), HBME-1 (37, 38), WT1 (4, 7, 9, 29, 33, 39), GLUT1 and IMP3 (4, 15). Adding one or more of them to the panel may improve specificity and lineage cell classification accuracy.

Although not a restriction for the principal aim of the study (comparison of the results between FC and IHC), the lack of a definitive histopathologic diagnosis represents a main limitation of our study, hampering the assessment of the diagnostic value of the panel. However, by integrating the results of this study with the current available literature, a possible diagnostic algorithm to interpret a panel including CK, VIM and DES in FC is described in Supplementary Figure 3. Prospective studies based on histopathologic diagnoses on a larger cohort of cases are needed to investigate its application and revisions based on future investigation of markers of reactive and neoplastic mesothelial cells are warranted.

In conclusion, our results show that FC can be a timesaving and multiparametric alternative to IHC on cell blocks in clinical settings. Histopathology of the primary lesion and immunohistochemistry should still be considered the main tools for a definitive diagnosis. However, the described method is an effective and non-invasive technique to refine the cytological diagnosis and can be easily integrated into routine panels to diagnose and characterize hematopoietic disorders. The FC and IHC interpretation of the panel is similar in most cases; however, occasional discordant results, particularly for VIM, may occur. A larger cohort of cases with histologic diagnosis is needed to evaluate the diagnostic accuracy of this technique and of the proposed algorithm. Being FC a flexible method that guarantees multiparameter analysis, the development of a multicolor approach and the inclusion of additional markers can improve and consolidate the panel.

## Data Availability

The raw data supporting the conclusions of this article will be made available by the authors, without undue reservation.
