# Supplementary material for: Flow cytometry of non-hematopoietic cells in canine effusions
Source: Front Vet Sci. 2024 Sep 24;11:1414271. doi: 10.3389/fvets.2024.1414271 (PMC11458718; doi:10.3389/fvets.2024.1414271)
Supplement: Supplementary file 1 [file Table_1.docx]

**Table. S1.** Routine fluid analysis results, cytological classification of non-hemopoietic (NH) cells, flow cytometric results, immunohistochemistry on cell blocks, final diagnosis based on clinical, pathological and follow-up data, and expected NH cell type of individual cases based on the final diagnosis.

|  |  |  |  |  | **FC** | | | | **IHC** | | |  |  |
| --- | --- | --- | --- | --- | --- | --- | --- | --- | --- | --- | --- | --- | --- |
| **CASE** | **Effusion** | **TP (g/dL)** | **TNCC (n/uL)** | **Cytological classification** | **NH cells (%)** | **CK (%)** | **VIM (%)** | **DES (%)** | **CK**  **(%)** | **VIM**  **(%)** | **DES**  **(%)** | **Final diagnosis** | **Expected cell type of predominant NH cells in effusion** |
| 1 | Pericardial | 4,30 | 14210 | mesothelial-doubtful | 13,3 | 87 | 9,8 | 71,9 | 78 | 85 | 76 | Idiopathic pericarditis | M |
| 2 | Pericardial | 2,90 | 2430 | mesothelial-reactive | 4,9 | 91,5 | 18,2 | 56 | 87 | 70 | 84 | Hemorrhagic pericardial effusion due to ruptured auricular mass (e.g., hematoma, hemangioma, hemangiosarcoma) | M |
| 3 | Peritoneal | 4,00 | 440 | mesothelial-reactive | 6 | 96,7 | 91,5 | 86 | 90 | 100 | 90 | Ascites secondary to acute cardiac tamponade | M |
| 4 | Pleural | 1,90 | 3340 | epithelial | 8,2 | 98,4 | 52,6 | 16,9 | 97 | 72 | 15 | Primary lung carcinoma | E |
| 5 | Pleural | 2,00 | 29260 | epithelial | 15,2 | 92,7 | 17,4 | 1,2 | 90 | 32 | 0 | Primary lung carcinoma | E |
| 6 | Pleural | 2,30 | 6010 | neoplastic-doubtful | 26,5 | 0,3 | 98,9 | 0,1 | 0 | 100 | 0 | Osteosarcoma | S |
| 7 | Peritoneal | 3,60 | 840 | mesothelial-reactive | 10,1 | 96,5 | 3,9 | 80 | 100 | 84 | 92 | Ascites secondary to atrial fibrillation | M |
| 8 | Pleural | 3,20 | 19210 | neoplastic-doubtful | 52,3 | 99,5 | 1,8 | 2,3 | 100 | 15 | 8 | Lung carcinoma | E |
| 9 | Peritoneal | 3,40 | 30910 | neoplastic-doubtful | 4,6 | 91,7 | 91,5 | 4 | 89 | 86 | 5 | na | na |
| 10 | Pericardial | 4,00 | 64650 | mesothelial-doubtful | 31,8 | 45,3 | 95 | 6,9 | 82 | 100 | 10 | Chronic fibrinous ulcerative lymphoplasmacytic pericarditis* | M |
| 11 | Pericardial | 3,80 | 19690 | mesothelial-doubtful | 12,9 | 96,9 | 37,5 | 50,1 | 98 | 70 | 64 | Idiopathic pericarditis | M |
| 12 | Pleural | 1,50 | 420 | mesothelial-reactive | 1,3 | 82,8 | 72,1 | 70,5 | 95 | 94 | 85 | Idiopathic lymphoplasmacytic pleuritis* | M |
| 13 | Pericardial | 5,80 | 15210 | mesothelial-reactive | 1,1 | 87 | 90,1 | 52,8 | 90 | 98 | 78 | Hemorrhagic pericardial effusion due to ruptured auricular mass (e.g., hematoma, hemangioma, hemangiosarcoma) | M |
| 14 | Pleural | 3,60 | 2020 | neoplastic-doubtful | 4,8 | 93,7 | 15,5 | 18,2 | 88 | 0 | 12 | Lung adenocarcinoma* | E |
| 15 | Pericardial | 4,80 | 12620 | mesothelial-doubtful | 20,2 | 97,3 | 21,4 | 90,4 | 100 | 82 | 80 | Idiopathic pericarditis | M |
| 16 | Peritoneal | 3,40 | 17550 | mesothelial-neoplastic | 1,6 | 76 | 53,8 | 6,6 | 88 | 84 | 14 | na | na |
| 17 | Pleural | 3,40 | 1010 | mesothelial-doubtful | 3 | 84,9 | 68,8 | 54,1 | 95 | 88 | 72 | na | na |
| 18 | Pleural | 3,00 | 4100 | neoplastic-doubtful | 65,3 | 91,5 | 10,8 | 2,2 | 90 | 12 | 2 | Lung carcinoma | E |
| 19 | Pleural | 3,80 | 25800 | epithelial | 18,1 | 96,9 | 5,5 | 0,9 | 100 | 100 | 3 | Disseminated mammary carcinoma |  |
| 20 | Pleural | 3,00 | 10770 | neoplastic-doubtful | 25,3 | 71,1 | 59,4 | 10 | 99 | 98 | 8 | na | na |
| 21 | Peritoneal | 2,80 | 36460 | neoplastic-doubtful | 8,4 | 91,2 | 93,1 | 4 | 100 | 100 | 15 | na | na |
| 22 | Pleural | 3,80 | 5850 | mesothelial-reactive | 14,2 | 72 | 63,7 | 47,5 | 90 | 95 | 70 | Idiopathic pleuritis | M |
| 23 | Peritoneal | 2,00 | 34310 | epithelial | 10,6 | 78,9 | 9,6 | 3,6 | 100 | 90 | 5 | Disseminated mammary carcinoma | E |
| 24 | Pleural | 2,20 | 5750 | neoplastic-doubtful | 8,7 | 97,7 | 43,2 | 96,2 | 91 | 89 | 82 | na | na |
| 25 | Pleural | nd | 34150 | epithelial | 23,3 | 99 | 0,4 | 5,9 | 92 | 0 | 0 | Mammary adenocarcinoma | E |
| 26 | Pleural | nd | 5810 | mesothelial-neoplastic | 42,2 | 98 | 1,6 | 34 | 98 | 10 | 15 | na | na |
| 27 | Pleural | 4,20 | 14190 | epithelial | 8,9 | 97,8 | 10,3 | 17 | 85 | 0 | 0 | na | na |
| 28 | Pleural | nd | 23220 | mesothelial-neoplastic | 49,5 | 93,2 | 1,5 | 86,6 | 78 | 78 | 25 | na | na |
| 29 | Peritoneal | nd | 164830 | mesothelial-neoplastic | 46,4 | 86,3 | 33,5 | 87,3 | 100 | 98 | 82 | Carcinoma | E |
| 30 | Peritoneal | nd | 3800 | epithelial | 30,1 | 97 | 4,1 | 10 | 98 | 0 | 0 | na | na |
| 31 | Peritoneal | 3,00 | 5230 | neoplastic-doubtful | 5,8 | 97,4 | 97,4 | 84,2 | 80 | 85 | 70 | Carcinoma | E |
| 32 | Pleural | nd | 21141 | epithelial | 15,3 | 98,9 | 12,5 | 4,5 | 95 | 0 | 0 | Carcinoma | E |
| 33 | Peritoneal | nd | 3700 | epithelial | 18,8 | 81,5 | 3,5 | 1,4 | 95 | 21 | 0 | Gastric carcinoma* | E |
| 34 | Pleural | 4,00 | 4430 | mesothelial-reactive | 8,7 | 90,1 | 54,4 | 94,1 | 72 | 85 | 50 | na | na |
| 35 | Peritoneal | nd | 30438 | epithelial | 15,7 | 97,9 | 5,9 | 3,1 | 75 | 5 | 0 | na | na |
| 36 | Pleural | 3,60 | 7830 | epithelial | 1,9 | 97,5 | 18 | 15,1 | 86 | 7 | 0 | na | na |

TP = total protein; TNCC = total nucleated cell count; FC = flow cytometry; IHC = immunohistochemistry on cell blocks; CK = cytokeratin; VIM = vimentin; DES = desmin; na = not applicable; * = histological diagnosis; M = mesothelial; E = epithelial; S = mesenchymal.
