# Supplementary material for: Flow cytometry of non-hematopoietic cells in canine effusions
Source: Front Vet Sci. 2024 Sep 24;11:1414271. doi: 10.3389/fvets.2024.1414271 (PMC11458718; doi:10.3389/fvets.2024.1414271)

Supplementary Figure S1. CD45-AlexaFluor647 vs CD11b-PerCP-eFluor710 labeling of a canine effusion. The three plots show the whole populationafter exclusion of doublets and events smaller than lymphocytes. A) A double-negative population is present (gate P4). B-C) Red color shows that CD45-negative events (B) correspond to large CD11b-negative events (C).

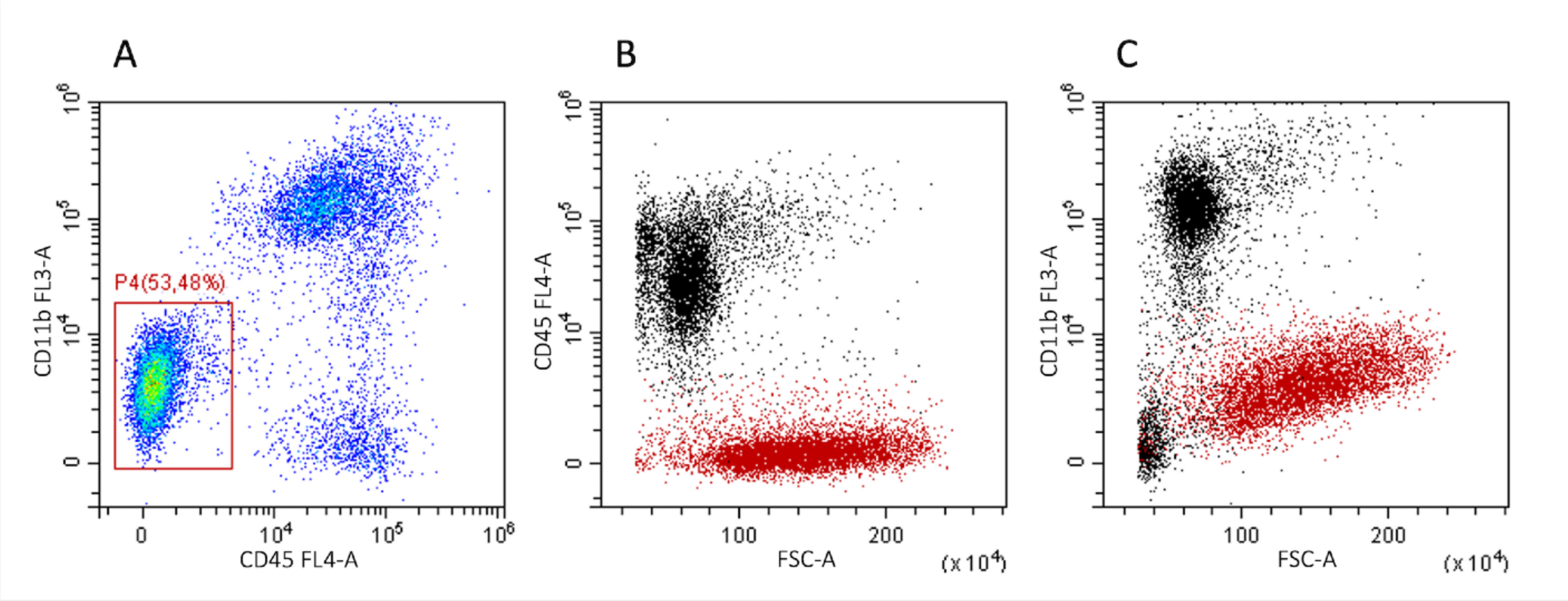

Supplement: Supplementary file 2 [file Data_Sheet_1.pdf]
