# Supplementary material for: Flow cytometry of non-hematopoietic cells in canine effusions
Source: Front Vet Sci. 2024 Sep 24;11:1414271. doi: 10.3389/fvets.2024.1414271 (PMC11458718; doi:10.3389/fvets.2024.1414271)

Supplementary Figure S2. Gating strategy. A) Gate P1 was set on FSC-A vs FSC-H plot to include singlets and exclude doublets. B) Only events in P1 are displayed. Gate P2 was set on FSC vs SSC plot to exclude events smaller than lymphocytes. D) Only events in P2 are displayed. Gate P3 was set on FSC vs CD45 to include only CD45-negative events. D) Only events in P3 are displayed. H1-UR quadrant was set on FSC vs FL1 to include less than 1% of events in the control tube.

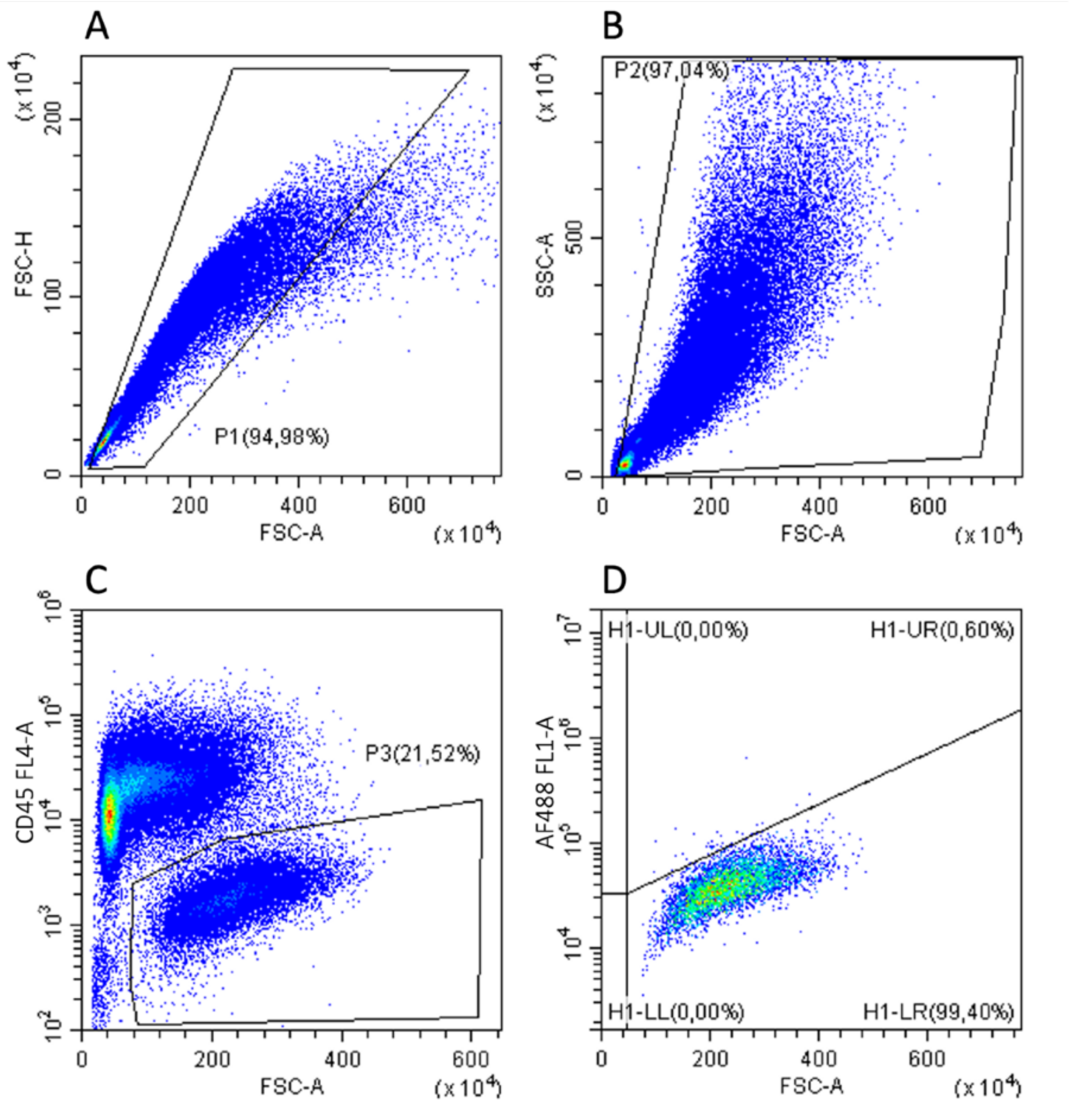

Supplement: Supplementary file 3 [file Data_Sheet_2.pdf]
