# Supplementary material for: Flow cytometry of non-hematopoietic cells in canine effusions
Source: Front Vet Sci. 2024 Sep 24;11:1414271. doi: 10.3389/fvets.2024.1414271 (PMC11458718; doi:10.3389/fvets.2024.1414271)
Supplement: Supplementary file 4 [file Data_Sheet_3.pdf]

Supplementary Figure S3. Proposed algorithm to interpret a panel including cytokeratin, vimentin and desmin to characterize non-hematopoietic cells by flow cytometry in canine effusions. The panel is designed to distinguish mesothelial and epithelial cells. It is assumed that the sample contains cells suspected to be of non-hematopoietic origin and the analysis is restricted to CD45-negative or large CD11b-negative target cells. target cells.

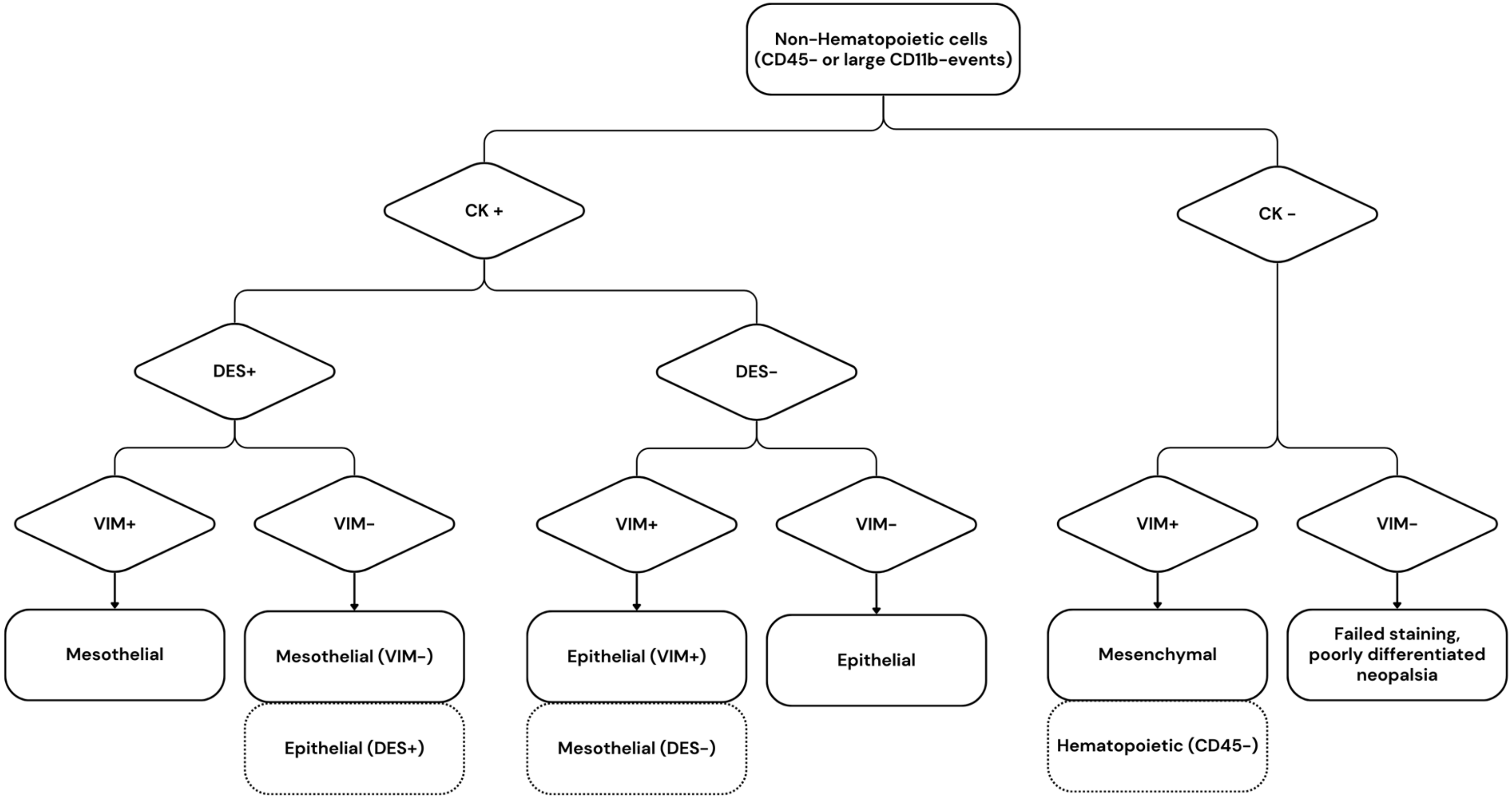

CK = cytokeratin; VIM = vimentin; DES = desmin; dotted boxes indicate a possible, but uncommon, alternative interpretation.
